# Case Report: Performing a Medication Safety Review Assisted by Pharmacogenomics to Explain a Prescribing Cascade Resulting in a Patient Fall

**DOI:** 10.3390/medicina59010118

**Published:** 2023-01-06

**Authors:** Joshua Russell, Meghan J. Arwood, Nicole M. Del Toro-Pagán, Nishita S. Amin, Michele D. Cambridge, Jacques Turgeon, Veronique Michaud

**Affiliations:** 1 Tabula Rasa HealthCare, Office of Translational Research and Residency Programs, Moorestown, NJ 08057, USA; 2 Tabula Rasa HealthCare, Precision Pharmacotherapy Research and Development Institute, Orlando, FL 32827, USA; 3 Franciscan Senior Health and Wellness, Program of All-Inclusive Care for the Elderly (PACE), Indianapolis, IN 46259, USA; 4 Faculty of Pharmacy, Université de Montréal, Montréal, QC H3C 3J7, Canada; 5 Faculty of Pharmacy, University of Montreal Hospital Research Center (CRCHUM), Montréal, QC H2X 0A9, Canada

**Keywords:** pharmacogenomics, pharmacogenetics, phenoconversion, prescribing cascade, fall, antidepressants, opioids, case report

## Abstract

Pharmacotherapy for major depressive disorder (MDD) typically consists of trial-and-error and clinician preference approaches, where patients often fail one or more antidepressants before finding an optimal regimen. Pharmacogenomics (PGx) can assist in prescribing appropriate antidepressants, thereby reducing the time to MDD remission and occurrence of adverse drug events. Since many antidepressants are metabolized by and/or inhibit cytochrome P450 enzymes (e.g., CYP2C19 or CYP2D6), drug-induced phenoconversion is common in patients on antidepressant combinations. This condition influences the interpretation of a patient’s PGx results, overall risk of ineffective/adverse medication response due to multi-drug interactions, and the recommendations. This complex case describes a patient with MDD, generalized anxiety disorder, and chronic pain who experienced a fall due to excessive sedation following a prescribing cascade of fluoxetine, bupropion, and doxepin. These antidepressants delivered a significant additive sedative effect and interacted with the patient’s hydrocodone, potentially contributing to uncontrolled pain, upward dose titration of hydrocodone, and a higher overall sedative burden. The PGx results and drug-induced phenoconversion described in this case report explain the patient’s excessive sedation and possibly ineffective/toxic antidepressant and opioid treatment. This case report also illustrates how a more timely multi-drug interaction assessment (preferably in conjunction with preemptive PGx testing) may have informed a different prescribing pattern, reduced/avoided a prescribing cascade, and potentially prevented a drug-related fall.

## 1. Introduction

As the population continues to age, the use of multiple medications is increasing at an alarming rate, with some reports showing an average of 14 or more prescriptions being taken by adults ≥50 years of age [1,2]. The comorbidities that increase with aging often necessitate polypharmacy, which increases drug-related iatrogenesis for a population that is at high risk for health problems [1,3]. Pharmacogenomics (PGx) assists in reducing medication-related iatrogenesis and optimizing medication regimens by identifying genomic variants known to impact drug disposition and response [3,4]. Clinicians can use PGx test results to guide dose/drug selection, along with consideration of other patient-specific factors (e.g., age, comorbidities, concomitant medications) [4]. This personalized approach must also consider phenoconversion—the condition defined as a mismatch between an individual’s genotype-predicted phenotype and their clinically observed phenotype [5]. Multiple factors can contribute to phenoconversion. The case described here explores drug-induced phenoconversion, which can be caused by non-competitive inhibition (e.g., via a cytochrome P450 [CYP] enzyme inhibitor binding to a CYP allosteric site, causing a conformational change in the active site and loss of affinity for the substrate), competitive inhibition (via a stronger affinity substrate given concurrently with a weaker affinity substrate), or induction (e.g., via an enzyme inducer) [5,6].

This case illustrates several occurrences of drug-induced phenoconversion, resulting in a prescribing cascade of multiple antidepressants and an opioid, and in an overall high sedative burden, which led to a fall-related injury. This prescribing cascade may have been influenced by pharmacotherapy failure due to drug–gene (DGIs) and drug–drug–gene interactions (DDGIs), contrasting the more commonly described prescribing cascade that results from misdiagnosis or treating an adverse drug event as a new condition [7,8]. Following the fall, the PGx clinical service was consulted, and PGx testing was performed. As part of this consult, a clinical pharmacist assessed and provided guidance on the patient’s PGx test results, with assistance from MedWise^®^, a clinical decision support system (CDSS). Utilizing the CDSS, the pharmacist can easily identify drug–drug interactions (DDIs) and possible drug-induced phenoconversion. The CDSS identifies non-competitive inhibitors (denoted in red), as well as substrates—the latter being further categorized as weak, moderate, or strong based on their affinity for the specific CYP enzyme (denoted in yellow, light orange, or dark orange, respectively) [9]. A competitive inhibition may occur when two drugs that are substrates for the same enzyme are coadministered. The magnitude of the competitive inhibition is a function of the affinity of the two substrates for the enzyme. This case provides support for the value of a comprehensive medication-safety review alongside PGx-guided prescribing which, had both been performed earlier, may have potentially avoided the prescribing cascade and resultant fall-related injury.

## 2. Description of the Case Report

**Figure 1:**
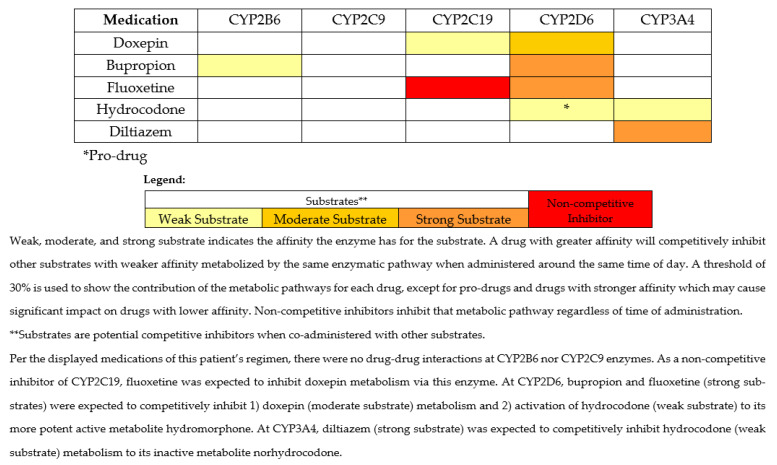
Metabolic pathways for medications of interest and interacting medications at the time of the fall.

In June 2020, a 59-year-old community-dwelling Caucasian female with a medical history of major depressive disorder (MDD), generalized anxiety disorder (GAD), chronic pain, and other diagnoses (Table 1) presented to her outpatient care facility after experiencing a fall, which resulted in a knee injury after she fell asleep on the toilet. At the time, the patient was prescribed fluoxetine, bupropion, and doxepin for MDD and hydrocodone for chronic pain. Duration, dosing/titration, and further details regarding this regimen prior to her care being established at this clinic were not available. The patient was diagnosed with unclassified chronic pain and pain due to cervical radiculopathy. Her hydrocodone dose had been increased during the previous year due to inadequate pain relief. The patient was taking her hydrocodone as prescribed by her outpatient facility, and prescriptions were filled in batches every 30 days. Further, there was no documented history of substance abuse. Other medications evaluated due to their sedating and fall-inducing properties were diltiazem, gabapentin, glipizide, and baclofen, which the patient had been taking at stable doses for greater than a year. The patient’s medications and their pathways of metabolism per the CDSS are described in Table 1 and Figure 1, respectively.

**Figure 2:**
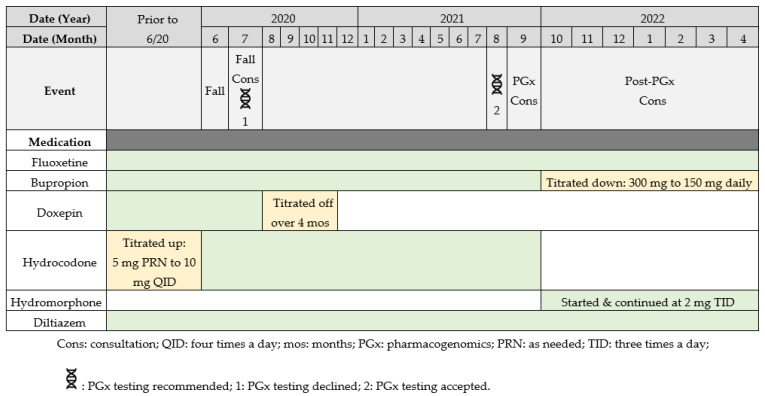
Timeline of events and medication changes.

The fall prompted a pharmacist-led fall consult to identify any offending medications. During the fall consult, the pharmacist recommended tapering the patient off doxepin (due to its high sedative burden value), which the provider accepted. Additionally, the pharmacist recommended PGx testing, as the results could inform the appropriateness of the patient’s antidepressant and opioid therapy. The provider initially did not accept this recommendation for testing; however, the provider accepted it about a year later in August 2021, when the pharmacist recommended testing for the second time as a part of a fall reduction initiative. A DNA sample was collected via buccal swab and analyzed by a Clinical Laboratory Improvement Amendments certified laboratory (OneOme, Minneapolis, MN, USA). The entire list of gene variants analyzed by the PGx provider is available on the OneOme website (https://oneome.com/gene-details/ [accessed on 8 September 2021]) [10]. The PGx results and a timeline of events regarding medication changes are depicted in Table 2 and Figure 2, respectively.

## 3. Pharmacist Recommendations

In September 2021, the clinical pharmacist performed a comprehensive review of the PGx results using the CDSS, assessing the overall and per-medication sedative burden as previously described [11], DDIs, DGIs, DDGIs, and other patient-specific factors (e.g., clinical response) to prepare recommendations for the provider. The CDSS revealed a high sedative burden value due to the opioid, two antidepressants, gabapentin, and baclofen in the medication regimen, illustrating the patient’s continued risk for oversedation. Considering the patient’s fall the year before and multi-DDIs, DGIs, and DDGIs, medications contributing to sedation became the focal point of the recommendations, which included medications contributing to management of the patient’s pain/MDD. The emphasis was to decrease the sedative load, while also optimizing the patient’s antidepressant and opioid therapy.

Following a review of the PGx test results while using the CDSS (depicted similarly to that in Figure 1, only without doxepin), the pharmacist conducted a medication safety review and suggested one of the following recommendations: (1) taper off bupropion and re-trial fluoxetine monotherapy or (2) switch bupropion to an alternative agent (e.g., desvenlafaxine or duloxetine), given the CYP2B6 poor metabolizer (PM) status. The pharmacist also recommended—if opioids were warranted—to consider switching hydrocodone to an opioid not metabolized by CYP2D6, such as hydromorphone, due to the multiple interactions at CYP2D6 contributing to the patient’s insufficient analgesic effect.

The recommendation to re-trial fluoxetine monotherapy was accepted, as the patient’s MDD and GAD symptoms had improved since the fall and the clinical team suspected that a single antidepressant may be sufficient for symptom control. Because the patient’s GAD symptoms remained the primary concern and her bupropion was predicted to have reduced efficacy based on PGx results, the provider reduced the dose from 300 mg to 150 mg daily, with the intention to eventually discontinue.

Hydromorphone was started with a 40% morphine milliequivalent (MME) dose reduction at 2 mg three times daily, since CYP2D6 drug-induced phenoconversion to potential PM status warranted consideration of the patient as opioid naïve, regardless of how long she had been on hydrocodone. Therefore, the higher than usual preemptive dose reduction was appropriate.

On follow-up call, approximately 8 months after the acceptance of recommendations, the patient has remained on hydromorphone at 2 mg three times daily with a reported improvement in pain control according to her provider. Additionally, the patient has experienced no additional falls and no longer shows signs of drowsiness/dizziness since the implementation of the recommendations.

## 4. Discussion

Drug-induced phenoconversion is common in older adults with polypharmacy being treated for MDD, as many antidepressants (e.g., selective serotonin reuptake inhibitors [SSRIs]) are metabolized by or interact with CYP2C19 and/or CYP2D6 enzymes [12,13,14]. For example, drug-induced phenoconversion can be observed if a patient identified as a CYP2C19 normal metabolizer (NM) takes fluoxetine, a CYP2C19 inhibitor, causing the observed phenotype to resemble a PM for the victim drug. PGx testing to identify CYP2C19 and CYP2D6 genetic variants that can alter pharmacokinetic parameters has been associated with improved efficacy outcomes with SSRIs [15,16,17]. Currently, the typical treatment approach of MDD and GAD is trial-and-error and clinician preference/experience, and individuals often experience long periods of inadequate management of depression/anxiety symptoms and/or adverse drug events before finding the optimal regimen [18,19]. PGx can aid in narrowing down antidepressant drug selection/dosing, contributing to improvements in response and faster remission and/or reduced risk of antidepressant-related toxicity [16,20,21,22]. The Clinical Pharmacogenetics Implementation Consortium (CPIC) and the Dutch Pharmacogenetics Working Group (DPWG) provide genotype/phenotype-based therapeutic recommendations for drug dosing/selection for SSRIs [17,23], serotonin-norepinephrine reuptake inhibitors [23], and tricyclic antidepressants (TCAs) [23,24].

In addition to demonstrating a role in depression pharmacotherapy, PGx has gained traction in the treatment of pain, especially with CYP2D6 and opioids. PGx-guided prescribing for opioids may improve response rates, reduce adverse drug reactions, increase adherence to analgesic treatment plans, and reduce opioid consumption without compromising pain control [25,26,27]. To guide the prescribing of opioids based on CYP2D6 results, CPIC and DPWG provide genotype/phenotype-based therapeutic recommendations [23,28]. Similar to what is seen in patients taking antidepressants, drug-induced phenoconversion is also frequently observed in patients who take opioids, highlighting the need for education surrounding drugs and drug classes that commonly cause or suffer from phenoconversion, as well as strategies on how to identify and ameliorate any negative effects from DDGIs [12,25]. Among all CYP genes, DDGIs are most common at CYP2D6, which are amplified in our elderly population due to a greater number of comedications and substrates inhibiting CYP2D6 [12]. Common medications that may cause phenoconversion at a CYP2D6-metabolized opioid (i.e., codeine, tramadol, hydrocodone, oxycodone) include paroxetine, fluoxetine, mirabegron, bupropion, and duloxetine [12,29]. In a retrospective analysis within the Veterans Health Administration that assessed trends in opioid prescribing and potential for DGIs and DDGIs over a 6-year period, opioids were co-prescribed with antidepressants interacting with CYP2D6 in 28% patients with at least one opioid prescription, with a significantly higher co-prescription rate (42%) among chronic opioid users [30]. Roughly 13% of the cohort was estimated to exhibit CYP2D6 DDGIs due to predicted NM/IM status and exposure to a moderate/strong CYP2D6 inhibitor [30], while CYP2D6 DDGI frequency has been shown to be as high as 25% or 40% in other practice settings utilizing CYP2D6-guided prescribing [12,29]. If left unaddressed, CYP2D6 DDGIs in individuals prescribed opioids may contribute to uncontrolled pain and unintentional misuse and/or overprescribing of opioids [26,27]. As described in this case, the provider increased the dose and frequency of opioid in response to CYP2D6 DDGI-related uncontrolled pain.

In this case, PGx provided insights into potential contributing reasons for the patient’s increased sedation with doxepin, apparent pharmacotherapy failures to bupropion and hydrocodone, and possible drug-related fall injury in a patient with an already high sedative burden. The patient was on three different sedating antidepressants, one being doxepin, a TCA that is metabolized by CYP2C19 (via demethylation) to its active metabolite, desmethyldoxepin (nordoxepin). Hydroxylation of both doxepin and nordoxepin by CYP2D6 leads to the clearance of the parent drug and the active metabolite [31]. Studies have shown that CYP2D6 PMs treated with doxepin have increased concentrations of both doxepin and nordoxepin compared to NMs [31]. Sedation is a known side effect of doxepin and other TCAs; for this reason, in the Beers Criteria, doxepin is considered potentially inappropriate for use in older adults [32]. As a result of having less functional CYP2C19 and/or CYP2D6 enzyme based on genotype (i.e., DGIs) and/or DDIs, higher concentrations of doxepin and nordoxepin are expected to increase risk of sedation and associated complications. In this case, the patient was a CYP2C19 rapid metabolizer (RM) and CYP2D6 intermediate metabolizer (IM), but fluoxetine and bupropion phenoconverted her to a possible PM for doxepin at both genes. The presence of drug-induced phenoconversion likely led to accumulation of doxepin, a highly sedative drug, contributing to her fall.

While doxepin is susceptible to drug-induced phenoconversion at CYP2D6, bupropion is a common cause of phenoconversion, especially in patients with concomitant MDD and pain [12,29,30]. Bupropion is primarily metabolized via CYP2B6 to hydroxybupropion (HB), an active metabolite that has about 50% the activity of the parent drug. However, plasma levels of HB are 10 to 30 times higher than bupropion under steady-state concentrations [33,34]. Given the much higher concentration of HB, it is likely to contribute the majority of the pharmacologic activity of bupropion. In vivo pharmacokinetic studies have shown that CYP2B6 PMs (e.g., CYP2B6 * 6/* 6) have lower steady-state HB/bupropion metabolic ratios and HB concentrations compared to NMs [33,35], the latter of which was corroborated in a recent meta-analysis [36]. Lower HB concentrations in plasma have been associated with reduced response to bupropion for depression [37]. For this reason, the reduction in CYP2B6 activity seen with CYP2B6 PMs likely increases the risk for therapeutic failure with bupropion. Case report evidence in a CYP2B6 PM taking bupropion illustrated a lack of efficacy with bupropion despite high doses [38]. Further, based on meta-analysis results showing a 33% decrease of HB area under the plasma drug concentration-time curve in CYP2B6 PMs, the investigators estimated that this reflected an approximately 50% higher bupropion dose requirement to achieve similar HB exposure as in NMs [36]. Recall that the patient in this case was a CYP2B6 PM and therefore was expected to exhibit less conversion to HB. This may have led to an inadequate/reduced response to bupropion despite optimized doses. While taking two additional antidepressants in conjunction with fluoxetine, the patient had an increased risk for pharmacotherapy failure while still having a high sedative burden. Despite no existing clinical guidelines for bupropion and CYP2B6, the MedWise^®^ CDSS tool guided the PGx pharmacist to assess this interaction for the patient, emphasizing a strength of this case. As such, primary literature was utilized to formulate antidepressant recommendations. Based on the assessment of the patient’s MDD and GAD symptoms, the provider decided which therapeutic option was best while considering the limited evidence regarding the impact of *CYP2B6* on response to bupropion. Limitations existed when making recommendations for antidepressant therapy. It was unclear if any other antidepressant therapies had been trialed prior to enrollment in clinical pharmacy services and in what order the antidepressants had been initiated.

In addition to the potential toxicity concerns exhibited with doxepin use and efficacy concerns with bupropion use, the patient also had a high risk of falls due to the DDIs affecting hydrocodone metabolism. Hydrocodone is metabolized by CYP2D6 to hydromorphone, a 2.5-times more potent mu opioid receptor agonist compared to hydrocodone, and by CYP3A4 to norhydrocodone, an inactive metabolite [39]. Given the patient’s predicted drug-induced phenoconversion to PM status by bupropion and fluoxetine, this patient likely had decreased concentrations of hydromorphone [40]. Lower concentrations of hydromorphone potentially contributed to poor pain control and led to a dose increase from 5 mg every 6 h as needed to 10 mg scheduled every 6 h. The DDIs present at CYP2D6 likely decreased the analgesic efficacy of hydrocodone, possibly explaining why the provider increased the hydrocodone dose over time. Regarding CYP3A4, this enzyme contributes to hydrocodone elimination producing an inactive metabolite [39]. Because this patient was taking diltiazem, which has a higher affinity for the CYP3A4 enzyme compared to hydrocodone, competitive inhibition would be expected, leading to decreased metabolism of hydrocodone to norhydrocodone [39]. This may have led to higher concentrations of hydrocodone and increased the risk of adverse drug effects associated with hydrocodone due to the metabolic pathway disruptions.

Reactive PGx testing, as used in our case above, was useful for post-fall medication regimen optimization, in conjunction with a thorough medication review. However, preemptive PGx testing, used when PGx test results are accessible prior to making treatment and dosing decisions [4], may have been beneficial in this case alongside the usual assessment of DDIs, as the combination may have prevented or reduced the burden of the prescribing cascade of multiple antidepressants. A preemptive PGx test may have better informed initial recommendations for MDD/GAD; had *CYP2B6* genotype been assessed preemptively, the medical team could have recognized that the patient may have an increased risk of inefficacy with bupropion and selected an alternative agent. However, given the literature for *CYP2B6* and bupropion is predominantly limited to pharmacokinetic studies and case reports [33,35,36,38], the provider might have been less inclined to accept such a recommendation.

In this case, the patient’s improved pain control after switching to hydromorphone (non-CYP2D6 opioid) revealed the value of a medication safety review that included reactive PGx testing. Further, pharmacist involvement led to decreasing dose/titration off bupropion and doxepin, which was determined to lower the patient’s sedative burden without compromising MDD/GAD control. These outcomes suggest that had a comprehensive medication safety review been performed sooner (ideally in conjunction with PGx testing), the patient may not have experienced the oversedation that led to the fall. Although the evidence for PGx use in depression [20,21,22] and pain [26,27] is compelling, much of it has been performed reactively. Arguments have been made in favor of preemptive PGx testing to guide the selection/dosing of antidepressants [16,22,41] and opioids [30,42] to provide more effective and safer therapy, and trials are underway that plan to shed more light on this [43,44].

## 5. Conclusions

Including PGx results in the clinical decision-making process alongside a thorough review of any potential DDIs may provide benefit to patients with chronic pain and/or depression. The medication safety review combined with PGx testing described in this case allowed for the identification of DGIs and DDGIs that likely resulted in additional drug accumulation and contributed to the patient’s oversedation and fall. Utilizing PGx test results and assessing for drug-induced phenoconversion has led to a reduction in sedative burden from antidepressants, adequate pain control, and no recurrence of falls in this patient. In this case, preemptive PGx-informed clinical-decision making may have streamlined the trial-and-error optimization of the patient’s antidepressants.

## Figures and Tables

**Table 1:** Medications at the time of the fall.

Medical Condition	Medication	Dose	Frequency
MDD/GAD	Bupropion HCl XL	300 mg	Daily
	Fluoxetine	40 mg	Daily
	Doxepin	75 mg	Nightly
Chronic Pain	Hydrocodone/acetaminophen	10 mg–325 mg	Four times daily
Muscle Spasms	Baclofen	10 mg	Every 6 h as needed
Asthma	Albuterol–Ipratropium	2.5–0.5 mg/3 mL	Every 6 h as needed
	Montelukast	10 mg	Nightly
Hyperlipidemia	Atorvastatin	80 mg	Nightly
Cardiomyopathy	Diltiazem	120 mg	Nightly
	Aspirin	81 mg	Daily
	Furosemide	40 mg	Daily
	Losartan	25 mg	Daily
DM	Metformin	1000 mg	Twice daily
	Sitagliptin	100 mg	Daily
	Glipizide	5 mg	Daily
Peripheral neuropathy	Gabapentin	800 mg	Four times daily
Hypothyroidism	Levothyroxine	50 mcg	Daily
Restless-Leg Syndrome	Pramipexole	1 mg	Three times daily
Vitamin deficiency	Calcium Carbonate	1250 mg	Daily
	Ferrous Sulfate	325 mg	Daily

MDD: major depressive disorder, GAD: generalized anxiety disorder, DM: diabetes mellitus.

**Table 2:** PGx results.

Gene	Genotype	Phenotype
*CYP2B6*	* 6/* 6	Poor Metabolizer (PM)
*CYP2C9*	* 1/* 1	Normal Metabolizer (NM)
*CYP2C19*	* 1/* 17	Rapid Metabolizer (RM)
*CYP2D6*	* 1/* 4	Intermediate Metabolizer (IM)
*CYP3A5*	* 3/* 3	Non-expresser

## Data Availability

The data presented in this case are available in the article.
